# Understanding DNA results within the case context: importance of the alternative proposition

**DOI:** 10.3389/fgene.2013.00242

**Published:** 2013-12-06

**Authors:** Louise McKenna

**Affiliations:** Justice, Forensic Science LaboratoryDublin, Ireland

**Keywords:** evidence evaluation, alternative proposition, pre-case assessment, DNA, nearby defence

## Introduction

There is a growing awareness of the dangers of reporting DNA profiling results in criminal investigations without consideration of the implications of the finding within the case context. A recent article in the New York Times (July 24 2013) described a robbery that resulted in the death of Raveesh Kumra. Foreign DNA on the victim's fingernails corresponded with the profile of a local man, Lukis Anderson who was charged with murder. Following 5 months in prison, it was found that Mr. Anderson could not have committed the crime as he was in hospital at the time of the robbery. This and other cases demonstrate that reporting the DNA profile results alone can be misleading. The investigators and courts may be impressed by the probity of the DNA result in isolation and not think about other issues such as the possibility of secondary transfer. The Association of Forensic Science Practitioners, UK and Ireland (2009) attempted to address this problem for trace evidence through the introduction of Standards for the Formulation of Evaluative Forensic Science Expert Opinion. These standards require that the scientific finding is considered relative to two mutually exclusive propositions, one from the prosecution and one from the defence [based on the work of Evett et al. (2000)]. Within the case context, the probability of the evidence given the prosecution proposition divided by the probability of the evidence given the defence proposition produces the Likelihood Ratio (LR). The magnitude of the LR indicates the degree of support for one proposition vs. the other. This approach allows the scientist to help the court to understand the implications of the findings for the particular circumstances of each case.

Since the publication of the AFSP standard, EFE (Forensic Science Laboratory, Dublin, Ireland) has worked at applying the criteria to its casework. The following examples are drawn from EFE casework and illustrate how the alternative proposition can significantly affect the impact of the DNA finding.

## Assault case

Mr. G was walking down a street in his home town when he was approached by three males. One of the men punched Mr. G, knocked him to the ground and kicked him a number of times in the head and chest area. Mr. G bled as a result of his injuries.

The police identified Mr. T as a suspect for this assault. They arrested Mr. T within 8 h of the incident and took his clothes and shoes. The laboratory found a single blood stain on Mr. T's jeans and the DNA profile corresponded with that of Mr. G. In the past, the EFE would have reported this observed correspondence as well as a probability assignment for the event that another unrelated person has the same DNA profile, in the order of less than one in a thousand million.

This is useful information if Mr. T claims that he had nothing to do with the incident and was not present when it occurred. But it could be misleading information if Mr. T has an explanation that results in a different alternative proposition.

In this case, Mr. T said he was one of the three men who approached Mr. G but he did not assault him. Mr. T says he ran away after the incident and never made contact with Mr. G. Therefore, the issue is whether Mr. T assaulted Mr. G or he was close by when the assault occurred. The appropriate propositions are:

Prosecution proposition: Mr. T punched and kicked Mr. G

Defence proposition: Mr. T was close by during the assault and someone else punched and kicked Mr. G

Prior to the examination of the clothes, the AFSP Standard requires scientists to consider their expectations for observing blood with a corresponding profile given these propositions. This is called the pre-case assessment. If Mr. T assaulted Mr. G, the scientist considers whether blood may or may not have transferred to Mr. T. For example, blood may not have transfered to Mr. T if the bleeding commenced after the assault ceased or if Mr. T's kicks did not make contact with the bloodstained area(s).

The scientist also considers the type of blood staining he would expect to observe given both propositions. For example, if Mr. T assaulted Mr. G, wet blood may have transferred to Mr. G's clothes or shoes as a result of contact. If Mr. T did not assault Mr. G but was nearby, then he is very unlikely to have made contact with a blood stained surface but airborne blood drops generated during the assault may have landed on his clothes. The trained scientist can usually distinguish contact from airborne stains as long as the stains are not so small, that smeared airborne stains could be confused with contact stains.

Using his/her understanding of how blood transfers in assaults, the scientist assigns probabilities for the presence of contact and/or airborne blood stains on the suspect's clothes given the two propositions (Table 1). They are not precise but help the scientist to understand that most outcomes (no blood, airborne blood stains, small contact stains) provide little assistance in the addressing the issue of whether Mr. T assaulted Mr. G or was close by during the assault. The exception is the presence of a large contact bloodstain or a number of small contact stains, which are unlikely if someone else assaulted Mr. G and Mr. T was close by. Therefore, it is worthwhile examining the clothes to see if this type of staining is present.

**Table 1:** **Pre-case assessment**.

**Outcomes on Mr. T's clothes**	**Probability of the finding given prosecution proposition**	**Probability of the finding given defence proposition**	***LR***
No blood with profile corresponding with Mr. G	0.5	0.8	0.63
Blood profile corresponds with Mr. G	0.5	0.2	2.5
Airborne only	0.05	0.15	0.33
Large contact/number of small contact blood stains that match Mr. G ± airborne	0.2	0.001	200
Small contact stain(s) that match Mr. G ± airborne stains	0.25	0.049	5

In this particular case, the single bloodstain found on Mr. T's jeans was an airborne stain. From the assigned probability for airborne blood stains and no contact stains (Table 1), the finding provides little assistance on the issue of whether Mr. T assaulted Mr. G or was standing nearby during the assault. However, if a large contact stain was found on Mr. T, then the reported conclusion would be that the finding provided moderately strong support for the proposition that Mr. T punched and kicked Mr. G rather than Mr. T was standing close by and someone else punched and kicked Mr. G.

The application of this type of logical reasoning demonstrates the importance of identifying the appropriate alternative. The previous practice of reporting the DNA result as a Conditional Profile Probability (CPP) without considering the case circumstances was at best unhelpful and could have been misleading. In fact, the CPP is of no value when the defence proposition allows for the presence of a corresponding profile. Another advantage is that the decision on the significance of different outcomes, before doing the examination, avoids the danger of *post-hoc* rationalization or bias on the part of the scientist.

## Firearm case

Police frequently submit firearms for DNA analysis and comparison with suspects. Take for example the situation where Mr. H was shot while driving his car through his gateway. A witness observed the shooter running to the get-away car and noted the registration number. A short time later, the police found the car. There had been an unsuccessful attempt to set the car on fire. A gun found in the car was submitted to the laboratory. Following their enquiries, the police identified Mr.M as a suspect. Mr. M says he had nothing to do with the shooting or the gun in question. The police requested that the laboratory examine the gun for DNA and if found to compare it with Mr. M's profile.

The laboratory got a single DNA profile from the gun and found that it did not correspond with Mr. M's profile. If they report this factually, will the police think that Mr.M should be excluded from their enquiries?

The propositions in this case are:

Prosecution proposition: Mr.M fired the gun.

Defence proposition: Mr.M had nothing to do with the gun, someone else fired it.

If Mr.M fired the gun, what is the probability of not finding his DNA (to) and finding somebody else's DNA as background (b)? Background DNA is defined as the interpretable DNA present on the gun that was not deposited by the person who last fired the gun.

If Mr.M did not fire the gun and some one else fired it, what is the probability of finding DNA different to Mr. M? This is the probability that DNA transferred from the person who fired the gun (t) and there is no background present (b_o_) or the probability that the DNA did not transfer from the person who fired the gun (t_o_) and there is background DNA present (b). (For simplicity, the conditional probability of the non-matching DNA profile is omitted as this cancels out).

LR=tobbto+tob.

Polley et al. (2006) examined the transfer rates of DNA from shooter to gun and observed an association between the shooter and the DNA profile approximately 30% of the time. This is also supported by other studies on transfer of DNA following handling (Phipps and Petricevic, 2007). These studies suggest 0.3 as the probability for transfer of the shooter's DNA to the gun (t) and 0.7 for the probability that the shooter's DNA did not transfer to the gun (t_o_).

Assigning a probability for the occurrence of background DNA is more difficult. DNA results from firearms in EFE show that no profile was obtained for 26% of firearms, mixed profiles in 35% and single profiles in 24% of firearms (and the DNA on the remainder could not be interpreted). It can then be deduced that there is interpretable background DNA on all the guns with mixed profiles and on some of the guns with single profiles. Therefore, the approximate range for background DNA on guns is between 0.35 and 0.6 (b).

We now see that the presence of DNA on the gun that does not correspond with Mr. M's profile is only slightly more likely if he did not fire the gun than if he did, suggesting that it would be unwise for the police to eliminate Mr.M from their enquiries on the basis of the DNA exclusion alone.

The example also illustrates that the frequency of background DNA on firearms, rather than the CPP, is the information required to assist the police investigation.

## Conclusion

When forensic science laboratories limit their DNA statements to reports of matching or non-matching DNA, the investigator and courts are deprived of the scientist's understanding of body fluid transfer, DNA transfer, DNA persistence and presence as background. The Standards for the Formulations of Evaluative Forensic Science Expert Opinion give the scientist guidance on how to interpret his or her findings in order to better assist the investigator and the court.
